# P-992. Global Estimated CMV Seroprevalence by Age and Sex

**DOI:** 10.1093/ofid/ofae631.1182

**Published:** 2025-01-29

**Authors:** Cathy Lally, W Dana Flanders, Anne Dilley, John D Diaz-Decaro

**Affiliations:** Epidemiologic Research and Methods, LLC, Atlanta, Georgia; Epidemiologic Research and Methods, LLC, Atlanta, Georgia; Epidemiologic Research & Methods, LLC, Southport, North Carolina; Moderna, Inc., Potomac, Maryland

## Abstract

**Background:**

Cytomegalovirus (CMV) infection, while typically subclinical, is a lifelong latent infection that has been implicated in various adverse outcomes, including accelerated immunosenescence, increased cardiovascular risk, and cognitive decline. However, during pregnancy, CMV infection can cause severe complications in neonates infected *in utero* (congenital CMV [cCMV]). We present modeled age-, sex-, and country-specific CMV seroprevalence estimates.

**Figure 1.**
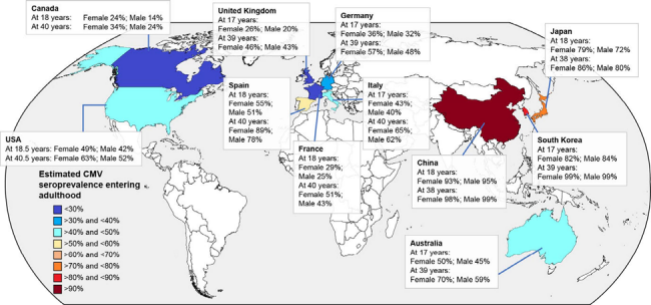
Modeled CMV Seroprevalence Estimates by Country, Age, and Sex

**Methods:**

PubMed and EMBASE searches were conducted in August 2022 to identify English-language literature, published 2005-2022, on CMV seroprevalence. Sex-specific seroprevalence by age category was extracted and smoothed using weighted regression; when ≥ 2 studies were identified, a random-effect meta-regression was used.

**Results:**

Overall, 25 studies were included. Estimated CMV seroprevalence increased with age and varied widely by sex and geographic location (**Figure 1**). Canada had the lowest seroprevalence estimates: 23.7% for females and 13.7% for males at age 18 years, and 33.5% and 23.5%, respectively, at age 40 years. The highest estimates at age 18 years were in China, with 93.0% for females and 95.3% for males, and at age 39 years in South Korea, with 99.1% and 98.8%, respectively. In Canada, France, Italy, Japan, UK, and USA, estimated seroprevalence was higher in females than in males across all age groups. In Australia, Germany, and Spain, estimated seroprevalence in younger age groups was higher among males than females, but in older age groups was higher among females than males. CMV seroprevalence was > 50% for both sexes in China at age 2 years, Japan at 4 years, and South Korea at 17 years, and for females at 14 years and males at 18 years in Spain.

**Conclusion:**

Modeled CMV seroprevalence estimates increased with age and were higher among females than males in most countries. The highest modeled CMV burden was observed among the Asian countries. This study highlights the seroprevalence of CMV among females during their reproductive years, with potential risk of cCMV in their unborn children. The findings underscore the urgent need for targeted strategies to mitigate the global burden of CMV, particularly among reproductive-age females.

**Disclosures:**

**Cathy Lally, MSPH**, Epidemiologic Research & Methods, LLC: Ownership Interest **W. Dana Flanders, MD, DSc, MPH, MA**, Epidemiologic Research & Methods, LLC: Ownership Interest **Anne Dilley, PhD**, Epidemiologic Research & Methods, LLC: Ownership Interest **John D. Diaz-Decaro, PhD, MS**, Moderna, Inc.: Employee|Moderna, Inc.: Stocks/Bonds (Public Company)

